# Making sense of fundal pressure: A qualitative study on women’s experiences of a non-evidence-based yet commonly practiced intervention

**DOI:** 10.1007/s00404-025-08130-3

**Published:** 2025-07-24

**Authors:** Mi-Ran Okumu, Lisa Bach, Ute Karbach, Lorna McKee, Florian Recker, Lissa Haid-Schmallenberg, Arno Stöcker, Anna Volkert, Nadine Scholten

**Affiliations:** 1 Center for Health Communication and Health Services Research, Department for Psychosomatic Medicine and Psychotherapy, Faculty of Medicine, University Hospital Bonn, Bonn, Germany; 2 Chair of Health Services Research, Faculty of Human Sciences and Faculty of Medicine and University Hospital Cologne, Institute of Medical Sociology, Health Services Research and Rehabilitation Science, University of Cologne, Cologne, Germany; 3 Chair of Quality Development and Evaluation in Rehabilitation, Faculty of Human Sciences and Faculty of Medicine and University Hospital Cologne, Institute of Medical Sociology, Health Services Research and Rehabilitation Science, University of Cologne, Cologne, Germany; 4 Emeritus Professor of Management and Health Services Research, Business School and School of Medicine, Medical Sciences and Nutrition, University of Aberdeen, Aberdeen, Scotland, UK; 5 Department of Obstetrics and Prenatal Medicine, University Hospital Bonn, Bonn, Germany

**Keywords:** Kristeller manoeuvre, Grounded theory, Obstetrics, Patient-centredness, Women-centredness, Non-evidence based interventions

## Abstract

**Introduction:**

Fundal pressure (FP) during second stage of labour has been discussed controversially. The intervention involves pressure to the uppermost part of the uterus to assist vaginal birth. While evidence is lacking, women report differing experiences from violent and traumatic to positive and helpful. This paper examines the experience of FP from the perspective of women without evaluating the intervention itself.

**Methods:**

The informed grounded theory study included 12 experiences of FP. The inclusion criteria were hospital births with application of FP no longer than 12 months ago. Inductively generated codes were aligned with a previously developed theoretical model on perception formation during obstetric situations.

**Results:**

The women’s appraisal of FP was determined by the level of perceived comprehensibility (understanding of situation and intervention) and manageability (ability to cope) as well as respective subcategories. Depending on the depictions of the participants, we determined six FP experiences as positive, three as neutral and three as negative. In all classifications, there were cases of low comprehensibility largely tied to brief medical explanations given the urgency of the situations. Regarding manageability, positive experiences were connected to high, neutral experiences to moderate, and negative experiences to low levels of manageability.

**Conclusion:**

Our study indicates that women’s appraisal of FP is determined by the level of comprehensibility and particularly manageability. In light of the controversies around FP, we do not take position whether FP should be applied or banned but conclude that if FP is applied, women’s comprehensibility and manageability need to be safeguarded.

**Supplementary Information:**

The online version contains supplementary material available at 10.1007/s00404-025-08130-3.

## What does this study add to the clinical work

**Table:** 

Despite lack of evidence and guidelines discouraging its practice, Fundal pressure (FP) is widely applied. If the intervention needs to be applied, women’s comprehensibility and manageability should be ensured through clear explanations, patient autonomy and ethical standards.	

## Introduction

Fundal pressure (FP), also known as Kristeller manoeuvre, is applied by obstetricians and midwives to assist vaginal birth and prevent protracted second stage of labour, instrumental birth or secondary caesarean section [1–5]. It involves manual pressure to the uppermost part of the uterus towards the birth canal [5]. To date, evidence regarding maternal and neonatal benefits and harms of the intervention is lacking [5, 6]. In this light, national and international guidelines advise against its use [7, 8] while some authors describe possibilities of gentle assisted pressure [9–11] and the need for standardised guidelines of application to prevent uncontrollable pressure and resulting harm [4, 12, 13]. The German obstetric guideline [14] recommends not to apply FP and to only consider it under strict medical indication and adherence to specific conditions such as the woman’s right of veto.

Beyond clinical debates, the application of FP raises ethical concerns regarding informed consent, patient autonomy and obstetric violence. Studies showed that interventions applied without explicit consent can contribute to negative birth experiences and long-term psychosocial consequences [15, 16]. Jardim and Modena [17] classify FP as obstetric violence (OV) and several studies underline the negative effects of the intervention on mother and child such as injuries, complications or posttraumatic stress [18–25].

Amidst the controversial debates and lacking evidence, FP continues to be applied worldwide [3, 6, 11, 26, 27], oftentimes undocumented [11, 28, 29] and in different methods of application [4, 30]. Existing literature mainly focuses on physical injuries [13, 20, 23] and the acceleration of birth process [29, 31] rather than examining how the intervention is implemented and communicated or how mothers experience it [5, 28, 32]. The limited number of studies describe varying experiences ranging from relieving and helpful to negative and even traumatic [18, 30, 32].

The main objective of this study is a better understanding of women’s experiences of FP. We seek to explore the influencing factors of the different appraisals while refraining from evaluating the intervention itself.

## Methods

The present study was conducted as part of MAM-Care, a mixed-methods study funded by the German Federal Ministry of Education and Research (FKZ: 01GY2110). Ethical approval was obtained from the Medical Faculty of the University of Cologne (vote no. 22–1260). We report in compliance with the consolidated criteria for reporting qualitative research (COREQ) [33]. Our data comprise interviews conducted by MO in 2023 as part of a previous Grounded Theory Methodology (GTM) study [34] within MAM-Care. Inclusion criteria involved women who had given birth in a hospital not longer than 12 months ago. The interviews captured various obstetric situations that the participants experienced from arrival at the delivery room until childbirth. The current analysis includes all interviews with experience of FP. We analysed 12 cases of FP from 11 interviews as one interviewee (ID10) had two contrasting experiences (ID10/1, ID10/2) applied by two different physicians. The interviewees were recruited through three calls for participation shared on the MAM-Care Instagram page^1^. The first and second recruitment post called for women who had experienced OV. The third post asked for women who had experienced FP, providing a neutral definition without mentioning OV. The interviews were guided by narrative prompts and follow-up questions. The first narrative prompt referred to the topic of the recruitment (experience of OV or FP). Additional narrative prompts and questions referred to further experiences during childbirth as well as expectations prior to childbirth. The interviews took place in the project office or through video call. Further details are described in the previous study [34]. Table 1 summarises the characteristics of the 11 interviewees. All interviews were conducted in German, all participants were native speakers.

**Table 1:** Characteristics of participants

Age at the time of delivery (mean; min–max)	
33; 27–38	
Parity	
Primiparous	10
Multiparous	1
Highest level of education	
Lower than A-levels	1
A-levels	2
University	8
Health insurance	
Statutory	8
Private	3

To explore the subjective experience of FP, we followed an informed GTM approach [37, 38]. To remain receptive to novel, unexpected aspects, we first derived themes and categories directly from the data (inductively). This was done by three female project researchers (MO, LB, LHS) with backgrounds in sociology, health education and midwifery. The themes and categories were regularly discussed with the wider team. Analysis and data collection were characterised by circularity and theoretical sampling as well as constant comparison. Data collection continued until no new themes emerged and theoretical saturation was achieved [35, 36]. The inductively derived categories were aligned to the categories of the previously developed theoretical model (deductively) on perception formation regarding obstetric situations [34].

According to the model, the appraisal of an obstetric situation is determined by the level of comprehensibility (understandability) and manageability (ability to cope with the situation). These two components were influenced by several subcategories as displayed in Table 2. The previous study revealed that in situations perceived as OV, participants felt low levels of comprehensibility and manageability while positive experiences were connected to high levels. The two components showed close interrelatedness, in fact, some subcategories influenced both components (overlaps).

**Table 2:** Category system

Sub-categories	Definitions of sub-categories	Categories
Comprehensibility of the situation	Cognitive understanding and physical intuition/sensing	Comprehensibility
Comprehensibility of the intervention	Cognitive understanding of what the intervention is about, how it is being exerted	
Sense of orientation	Availability of contact person (hospital staff)	Comprehensibility and manageability (overlaps)
Expectation in comparison to actual experience	Alignment of expectation (based on explanations, prior knowledge) and the experience	
Sense of trust	Interpersonal and professional trust in hospital staff	
Sense of control	Level of self-determination and self-efficacy during FP	Manageability
Physical sensation	Physical perception of FP	

Based on the establishment of the individual levels of each subcategory, we classified all FP experiences. For this purpose, we assessed narrations and expressions used by the participants to describe their experiences of the intervention (for example awful, pleasant). Mentioning only positive aspects was rated as positive (+), a mixture of positive and negative aspects as neutral (+/−) and only negative aspects as negative (−).

## Results

Twelve FP experiences were analysed. Table 2 provides an overview of the category system. In the previous study [34], comprehensibility was subdivided into cognitive and physical (intuition) comprehensibility. Due to the urgency of imminent FP and accordingly reduced medical information, in the current study, we distinguished between comprehensibility of the situation (both cognitively and physically) and of the intervention (cognitively).

Six experiences were classified as positive, three as neutral and three as negative. In all classifications, we found participants who had received epidural anaesthesia and others who did not. Further, different ways of execution (with hands, underarm, elbow, lateral upper body, using a cloth) were found in all classifications.

### Positive experiences

In this experience classification, high levels of situational comprehensibility referred to understanding the urgency of the situation, the need for an intervention as well as the feeling of being unable to push out the child on their own: *“It was urgent, […] because the fetal heart beats kept getting worse”* (ID10). “*I could actually feel clearly what needed to happen next: In addition to what I was physically able to do on my own, more pressure had to be applied for her [child] to come out”* (ID4).

Comprehensibility of the intervention often depended on explanations of the caregivers as described by interviewee 7: *“The senior doctor told me that during the next contraction she would like to press and push above the belly where the baby’s bottom is, to see if the baby stays in the pelvis or perhaps even moves towards the birth canal.”* Due to the urgency of the situation, explanations were generally depicted as brief and two interviewees could not understand the medical explanation: *“`I am going to push along’, no idea what he [physician] meant by that”* (ID11).

Interviewee 11 got scared after the midwife said that the intervention may be experienced as traumatising. Participants with prior knowledge mentioned that they only understood what the intervention was about due to this prior knowledge. In two cases, despite prior knowledge, participants could not recognise that FP was meant. *“To be honest, I didn’t realise it was FP until after birth”* (ID12). This was also related to the fact that in only three cases medical staff used the term FP or Kristeller manoeuvre to explain the intervention.

The participants described high levels of trust in the medical staff: *“Maybe that’s why I had this confidence, because I trusted the midwife* (ID12).” *"I knew that if the midwives suggested something […], that it would help me."* (ID4). Only interviewee 11 had a moderate level of trust. She disliked the physician, but consciously decided to embrace the situation and trust him.

The participants indicated that the intervention met their expectations or that they were pleasantly surprised: Interviewee 9 and 11 felt low comprehensibility but experienced the intervention as surprisingly pleasant.

Interviewees described high levels of control and underlined the perceived self-efficacy: *“If I had just been lying there while she applied FP during the contraction, I don’t think it would have worked so well. But by gathering all my strength and really giving it my all to push, I supported her [physician]. Actually the question is, did she support me or did I support her?”* (ID9). Further, interviewee 9 felt reassured by the midwife mentioning her right of veto.

In this classification, participants described that the intervention felt supportive and relieving and that they did not feel pain or pain did not play a prominent role. *“I only felt a sensation of pressure under the epidural, this was also the case during the execution of the Kristeller manoeuvre. I just had the feeling that I was helped to find the way down better. But there was no pain.”* (ID10/2) *“I really felt it as a gentle pushing along, […] like someone is helping to press something down, […] that I am not able to push down with my own strength”* (ID12).

In some cases, the high levels of comprehensibility and manageability were also influenced by specific preferences: Interviewee 4 and 10 wanted to avoid caesarean sections while interviewee 11 wanted to avoid a forceps delivery*. “I just wanted this child to be born vaginally, I didn’t want an emergency caesarean section.”* (ID10). None of the interviewees mentioned that caregivers gave alternatives to FP: *“I was not informed about alternatives. […] There was no ‘If we don’t do this, then we could do that.’ It was more like ‘Okay, we have to do this now, but you are allowed to say stop’”* (ID11). Table 3 gives an overview of the perceptions of comprehensibility and manageability with respect to positive experiences of FP.

**Table 3:** Positive experiences of FP

	C	M	Representative quotes regarding C (comprehensibility) and M (Manageability)
ID4	+	+	*One of the midwives, who was resting her elbow on my stomach, explained it in detail: ‘[…], I’m going to take my elbow, I’m going to lean on you heavily and I’m going to push really hard. Here we go.’ Well, I thought that was really quite remarkable that in a situation like this, which was stressful for everyone involved, everything was explained, and consent was asked for at all times. So it was absolutely clear to me the whole time that nothing would happen here to which I didn’t say ‘yes’*
ID7	+	+	*Well, she told me that very clearly. She showed me where the baby’s bum was, roughly on my tummy, and then she showed me where she was going to push. And then she said: ‘I would lean on there with my elbow and then try to push the little one down a bit with my elbow and then guide with my arm, with my hand, to somehow provide direction.’ And that’s exactly what she did.*
			*I didn’t find it unpleasant at all or more painful than the contractions without this grip, without this pushing. On the contrary, I actually found it beneficial and helpful. […] And I am grateful to her [physician] that she did it that way, because before [pushing alone] was just fruitless.*
ID9	–	+	*At first, I couldn’t really imagine what it [medical information] meant. It wasn’t until she demonstrated it during the contraction break that I understood what it actually meant and was a bit scared of it because it [the demonstration] was very unpleasant for me. But I thought: ‘Okay, if it helps me.’*
			*The doctor really made me feel comfortable and I had the feeling that if I said, ‘Stop, I don’t want it anymore’, she would really stop […] I really felt comfortable at that moment and thought: ‘Okay, I can trust her and as long as it’s okay for me, I would give it a try.’*
ID10/2	+	+	*He [senior physician] came in, you could tell he was in a hurry, he had only been in the room for like five or ten minutes, but he looked straight at me and said: ‘You’ve made it this far, we can do it now. Another one or two contractions. I’m going to help you again from the outside, if you don’t want it, then we’ll stop immediately, […] but I know you can do it.’ And he spoke to me so directly and encouraged me so much that I don’t think he even had to exert fundal pressure […].*
			*He [physician] knew exactly how to do it and it was exactly the right distribution of pressure. It wasn’t too much, and it wasn’t too little. […] it was a feeling of relief during birth.*
ID11	–	+	*At that time, I didn’t even know this form of intervention existed […]. And then the midwife said: ‘It may be unpleasant. The doctor is about to press on your belly and push along with you. Many women experience this as traumatising and if you feel this way, then we stop immediately.’ For a short time, the word ‘traumatising’ really scared me.*
			*The doctor pushed and my baby’s head dropped very low and from then on I was able to push and practically give birth to the baby on my own. In the end, I have to say that I am very grateful. I found it really pleasant because at last, something happened.*
ID12	+	+	*Then the gynaecologist asked, she would help or push along a little. […] somehow that felt absolutely right […].*
			*At that moment, I perceived the gynaecologist’s intervention as supportive. It was like a pushing along. I didn’t feel any pain, […] I was in a kind of pain bubble during every contraction anyway. That’s why I wouldn’t describe it [pain] as something outstanding.*

### Neutral experiences

In this experience classification, two participants described high levels of comprehensibility. One experienced low situational comprehensibility: She realised that the midwife became hectic but she herself did not see any urgency: “*There was no imminent danger for the child. This child had heartbeats, this child didn’t have an umbilical cord around its neck […], everything was fine”* (ID6). The midwife shouted at her to hold her breath when pushing and applied FP without prior communication. The participant highlighted that she only perceived the intervention marginally: *“I didn’t really pay attention to the pressure. I focused on pushing. I can’t really say if it [FP] was helpful or not because when she stopped, I kept pushing on my own and then the baby came.”* The strong focus on pushing and finally giving birth to the child on her own contributed to a higher level of control which led to a neutral level of manageability in spite of lack of trust and the unannounced intervention.

Interviewee 10 (ID10/1) narrated that an assistant physician unexpectedly held the child in place between the contractions which was uncomfortable and painful. Her veto being recognised immediately restored her sense of control and led to a neutral appraisal of the experience.

Interviewee 8 generally experienced low levels of control throughout the birth process: She could not feel the contractions well due to the epidural and because of foetal distress she only had few attempts to push before FP was applied. To her, FP was neither helpful nor negative. Both interviewee 6 and 8 did not mention exacerbated pain during FP. Table 4 shows the perceptions of comprehensibility and manageability in relation to neutral experiences of FP.

**Table 4:** Neutral experiences of FP

	C	M	Representative quotes regarding C (Comprehensibility) and M (Manageability)
ID6	–	+ –	*After the second or third push didn’t work, she [midwife] placed herself on me and pushed, she didn’t speak to me beforehand. She didn’t speak to me during FP either, she didn’t say what she was doing or ask if everything was okay. She just did her thing […].*
			*I can’t tell whether I found the pressure uncomfortable at the time, because I was so caught up in pushing.*
ID8	+	+ –	*At that moment, I knew what they meant [FP]. I had prepared myself for birth, what kind of interventions exist. I’d never seen a picture of how it [FP] actually happens. But I knew, okay, now there’s going to be some kind of pressure on my belly from above.*
			*It wasn’t physically unpleasant […].*
			*It was just so much at once, so many different things, so many people were there, but I didn’t think it was a big deal. It was just part of it, so I don’t see it as that negative. If it actually helped? It didn’t really [in the end interviewee 8 had an emergency caesarean section].*
			*For me, it was okay. You always hear those stories about it [FP] being traumatic, like the doctor throwing himself on your stomach. But that wasn’t how I experienced it at all. So, from that perspective, it was fine the way it went.*
ID10/1	+	+ –	*In the antenatal class we also talked about the controversies around the Kristeller manoeuvre […]. And because of that I had already thought about it before the birth actually started […]. And I think it was good to have a certain idea of what it was beforehand and to have dealt with it mentally. Well, I think if she had told me that without me knowing what it was about, I would have been surprised what she meant by ‘a little help’, because it wasn’t just a little, it was a real intervention.*
			*She then helped with the next contraction. But I felt that the pressure she exerted was too little and she then held the baby outside the contraction, she really pressed hard on my tummy, and I felt like she was squeezing the air out of me. That was a really unpleasant sensation at the moment when there was no contraction. And then I told her to stop, and she stopped immediately.*
			*I felt it [sense of control] the whole time, because she really stopped as soon as I said ‘I can’t do it like this and I can’t breathe’.*

### Negative experiences

In this experience classification, two participants experienced high levels of comprehensibility while one comprehended neither the situation nor the intervention wishing for more calmness and time. Although she had prior knowledge about FP, she did not recognise it from the medical explanation: “‘*We’re helping you, we’re helping to push from above’ […] I couldn’t understand that it was this [FP].”* Interviewee 3 who felt the urgency of the situation did not want a detailed explanation: “*I just thought: ‘I don’t care, get the child out’.”*

All interviewees of this classification experienced lack of coherence between expectation and experience. Interviewee 3 had a high level of comprehensibility but was overwhelmed by the sudden start of the intervention. Further, the intervention was exerted in an unexpected way. Interviewee 1 and 5 were overwhelmed by unexpectedly strong pain. Participants in this category described low levels of trust in the staff performing the intervention: *“The doctor who came in to perform the maneuver—I didn’t know her. I had no connection with her. She just came in and was terrible. […]”* (ID3).

The participants described low levels of control. Interviewee 5 narrated that her veto was ignored by the physician. Interviewee 3 felt powerlessly overwhelmed by the FP experience: *“My husband who is pressing my chin to my chest, someone pulling down there, the midwife shouting at me to push and someone sitting on top of me and pressing down.*” Table 5 summarises the perceptions of comprehensibility and manageability related to negative experiences of FP.

**Table 5:** Negative experiences of FP

	C	M	Representative quotes regarding C (Comprehensibility) and M (Manageability)
ID1	+	–	*I had heard about it that they support the birth process by pressing from above. But I had no idea how painful it would be.*
			*Above all it was this emotional violence [blaming], but also the physical violence when these two people, the midwife and the senior doctor, pushed the baby out.*
ID3	+	–	*My husband pushed my head down. The doctor was fiddling around with the suction cup and the other one started and pushed without saying another word.*
			*I knew this manoeuvre but had imagined it […] to be different from what happened. […] I had thought: ‘When I push the contraction downwards, someone will push with the contraction from above.’ […] They communicated everything openly, but I was just screaming because I didn’t know anymore: ‘What on earth is going on here?’ The moment they started, I was so focused on the next contraction. And then she started and really pushed like […] a resuscitation movement, […] she jumped up and down and I just didn’t know what to do with myself anymore. I was just screaming. I wasn’t pushing at all because at that moment I just thought: ‘What on earth is going on here?’ That was just too much.*
ID5	–	–	*For me, it wasn’t that urgent. […] I actually needed more time and more calmness.*
			*The announcement was like ‘I’ll push along a bit’ […]. By the time my head had even processed what that could mean, it had already happened.*
			*And then she [physician] did it [FP] the first time and it hurt immensely and was incredibly uncomfortable and I pushed her hands away and said: ‘No, I don’t want that.’ […] but then she repeated it a second time.*
			*That [FP] was the downside of the birth experience. […] Actually, it was not even the physical pain as such, but how I felt that I wasn’t seen and respected was the negative part.*

### Summary of the results

Across all experience classifications, high and low levels of comprehensibility were experienced. However, the perception of manageability varied greatly among the different experience classificaons (see table 6). Apart from sense of orientation, all subcategories related to manageability indicate that positive experiences are connected to high levels, neutral experiences to moderate levels and negative experiences to low levels. Although all participants had a high sense of orientation, meaning that they had an explicit professional contact person, they experienced different levels of trust and control.

**Table 6:** Summary of participants’ experiences of FP

Experience		Comprehensibility		Overlaps (Comprehensibility and Manageability)			Manageability	
		Comprehensibility of situation	Comprehensibility of intervention	Sense of Orientation	Trust	Expectation/Experience	Sense of Control	Physical sensation
Positive	ID4	+	+	+	+	+	+	+
	ID7	+	+	+	+	+	+	+
	ID9	+	–	+	+	+	+	+
	ID10/2	+	+	+	+	+	+	+
	ID11	+	–	+	+ –	+	+	+
	ID12	+	+	+	+	+	+	+
Neutral	ID6	–	–	+	–	+ –	+ –	+ –
	ID8	+	+	+	+	+	+ –	+ –
	ID10/1	+	+	+	+	-	+	–
Negative	ID1	+	+	+	–	–	–	–
	ID3	+	+	+	–	–	–	+ –
	ID5	–	–	+	–	–	–	–

## Discussion

The aim of this study was to explain the participants’ differing experiences of FP. For this purpose, we established their level of comprehensibility and manageability based on the previously developed theoretical model [34].

Although not recommended by the national obstetric guideline, FP is commonly used in German hospitals [27]. It takes place during second stage of labour and protracted birth processes which are per se vulnerable situations for the women as described in the literature [32, 39, 40]. Urgent situations have been referred to as challenging regarding medical safety, patient communication and involvement [41]. All participants described that caregivers perceived the situation as urgent. This was comprehended by most participants. However, participants with low situational comprehensibility perceived the caregivers as unnecessarily hectic. This indicates that patients and caregivers may interpret situations differently. Caregivers may expect women to understand what is going on and therefore not see the need to explain actions and interventions as shown in previous research [32, 42]. This underlines the need for continuous and more patient-centred communication.

Although the importance of clear preliminary explanations as well as obtaining consent before applying FP have been emphasised in the literature [4, 7, 14, 43], our study reveals room for improvement in this regard. Participants pointed out that medical explanations were brief and alternatives were not mentioned. In some cases, FP was not named but described as helping or pushing along while in one case the intervention took place without any communication. Such observations were also made by other studies on FP [11, 44]. While some participants received comprehensible explanations, others relied on prior knowledge or were left without understanding the intervention. This highlights a gap in patient-centred care. It may be useful to intensify training for caregivers in terms of effective communication in urgent situations. Future research should examine how individualised communication strategies could enhance maternal experiences of involvement and decision-making. From the perspective of mothers, it may be useful to address the intervention in prenatal classes since previous research has shown how prior knowledge can foster comprehending explanations, knowing preferences and participating in decision-making [45–47].

Our findings suggest that the perceived manageability plays a crucial role in shaping women’s experiences of FP. Manageability was high in positive experiences, moderate in neutral and low in negative experiences. In terms of physical sensation, in all positive experiences, participants could feel how the intervention helped them progressing. In the negative experiences, two participants stressed the strong pain but for one participant, the physical sensation was played out by the overwhelming loss of control. Such differing physical sensations of FP were also described in previous research [30, 32] which again calls for individualised patient-centred approaches. Within our sample, epidural anaesthesia did not seem to influence pain perception during FP.

Strikingly, comprehensibility showed mixed results and despite not understanding the intervention, some participants experienced FP positively. Notably, the level of manageability seemed to determine how the experience was appraised. This confirms our previous study [34], where we suggested that comprehensibility influences manageability but that the appraisal is mainly determined by the level of manageability. Experiencing the intervention as pleasant and helpful seemed to outweigh previous incomprehensibility. Notwithstanding, in the specific moment, incomprehensibility was experienced negatively which calls for more effective communication. In fact, previous research emphasised that lack of information can lead to lack of control and loss of autonomy [11, 19, 27] which in the end affects the level of satisfaction with the birth experience.

### Study limitations

The heterogeneous birth experiences and varying executions of FP may at first seem not analytically comparable. Yet, the presence of all variations of FP application across each experience classification supported a robust comparative qualitative analysis. Further, our focus was on the individual experience and subjectively striking aspects. Therefore, we did not specifically ask for obstetric or neonatal outcomes such as Apgar-score or complications postpartum. In future studies, it may be worthwhile to quantitatively compare the different experiences of FP against clinical data. Our participants were mainly native speakers with higher educational levels. Since we only considered the experience of FP in the situation, studies on long-term effects may be interesting.

## Conclusion

Our study examines how FP is experienced by women: The level of perceived comprehensibility and manageability influenced if FP was experienced as positive, neutral or negative. While our findings do not advocate for or against its use, they underscore the importance of ensuring that if FP is applied, the woman’s comprehensibility and manageability need to be safeguarded translating to clear explanations, patient autonomy and adherence to ethical standards. Strengthening patient-centred approaches may mitigate negative birth experiences associated with FP and other interventions. Obstetric training programmes should emphasise communication strategies that prioritise shared decision-making, particularly in high-stress birth situations even beyond FP. Additionally, we highlight the urgent need for clearer clinical guidelines on the application and communication of FP. Hospitals should establish transparent documentation practices for FP to ensure that the intervention is only applied when medically justified and to facilitate further research. The latter should also focus on the underlying reasons behind the common practice in spite of controversial discussions, lacking evidence and opposing guidelines.

## Supplementary Information

Below is the link to the electronic supplementary material.

Supplementary file2 (PDF 482 KB)

## Data Availability

Due to its sensitive nature and the assurance of anonymity, the data are not publicly available.

## Footnotes

1 https://www.instagram.com/mam.care/

## References

[1] Kline-Kaye V, Miller-Slade D (1990) The use of fundal pressure during the second stage of labor. J Obstet Gynecol Neonatal Nurs 19:511–517. 10.1111/j.1552-6909.1990.tb01670.x 2269906 10.1111/j.1552-6909.1990.tb01670.x

[2] Habek D, Vuković Bobić M, Hrgović Z (2008) Possible feto-maternal clinical risk of the Kristeller’s expression. Open Med 3:183–186. 10.2478/s11536-008-0008-z

[3] Farrington E, Connolly M, Phung L, Wilson AN, Comrie-Thomson L, Bohren MA et al (2021) The prevalence of uterine fundal pressure during the second stage of labour for women giving birth in health facilities: a systematic review and meta-analysis. Reprod Health 18:1–17. 10.1186/s12978-021-01148-1 34006288 10.1186/s12978-021-01148-1 PMC8132352

[4] Sagi-Dain L, Maymon R (2022) The condemned fundal pressure maneuver: time to reconsider? Arch Gynecol Obstet 306:1953–1957. 10.1007/s00404-022-06497-1 35277748 10.1007/s00404-022-06497-1

[5] Hofmeyr GJ, Vogel JP, Cuthbert A, Singata M (2017) Fundal pressure during the second stage of labour. Cochrane Database Syst Rev. 10.1002/14651858.CD006067.pub3 28267223 10.1002/14651858.CD006067.pub3 PMC6464399

[6] Furrer R, Schäffer L, Kimmich N, Zimmermann R, Haslinger C (2016) Maternal and fetal outcomes after uterine fundal pressure in spontaneous and assisted vaginal deliveries. J Perinat Med 44:767–772. 10.1515/jpm-2015-0101 26352067 10.1515/jpm-2015-0101

[7] World Health Organization (WHO) (2018) WHO redommentations intrapartum care for a positive childbirth experience: transforming care of women and babies for improved health and well-being. World Health Organization, Geneva

[8] Wright A, Nassar AH, Visser G, Ramasauskaite D, Theron G (2021) FIGO good clinical practice paper: management of the second stage of labor. Int J Gynaecol Obstet 152:172–181. 10.1002/ijgo.13552 33340411 10.1002/ijgo.13552 PMC7898872

[9] Novikova N, Mshweshwe N, Xoliswa W, Moloi P, Singata M, Hofmeyr J (2009) O688 a new method of controlled fundal pressure during the second stage of labour: Randomized pilot study. Int J Gynecol Obstet 107:S290. 10.1016/S0020-7292(09)61061-4

[10] Hofmeyr GJ, Vogel JP, Singata M, Habib NA, Landoulsi S, Gülmezoglu AM (2018) Does gentle assisted pushing or giving birth in the upright position reduce the duration of the second stage of labour? A three-arm, open-label, randomised controlled trial in South Africa. BMJ Glob Health 3(3):1–10. 10.1136/bmjgh-2018-000906 10.1136/bmjgh-2018-000906 PMC6035507 29989055

[11] Rubashkin N, Torres C, Escuriet R, Ruiz-Berdún MD (2019) “Just a little help”: a qualitative inquiry into the persistent use of uterine fundal pressure in the second stage of labor in Spain. Birth 46:517–522. 10.1111/birt.12424 30859644 10.1111/birt.12424

[12] Sagi-Dain L, Maymon R (2023) Fundal pressure: to avoid greater evil. Arch Gynecol Obstet 308:1911–1912. 10.1007/s00404-022-06894-6 36596988 10.1007/s00404-022-06894-6

[13] Bukovec P, Šturm B, Hodnik JJ, Drusany Starič K (2022) The influence of the fundal pressure manoeuvre at delivery on the anal sphincter injury diagnosed with endoanal ultrasonography. Eur J Obstet Gynecol Reprod Biol 273:65–68. 10.1016/j.ejogrb.2022.04.013 35504115 10.1016/j.ejogrb.2022.04.013

[14] Abou-Dakn M, Schäfers R, Peterwerth N, Asmushen K, Bässler-Weber S, Boes U et al (2022) Vaginal birth at term - part 2. Guideline of the DGGG, OEGGG and SGGG. Geburtshilfe Frauenheilkd 82:1194–1248. 10.1055/a-1904-6769 36339632 10.1055/a-1904-6769 PMC9633230

[15] Annborn A, Finnbogadóttir HR (2022) Obstetric violence a qualitative interview study. Midwifery 105:1–7. 10.1016/j.midw.2021.103212 10.1016/j.midw.2021.103212 34872035

[16] Vogels-Broeke M, Cellissen E, Daemers D, Budé L, de Vries R, Nieuwenhuijze M (2023) Women’s decision-making autonomy in Dutch maternity care. Birth 50:384–395. 10.1111/birt.12674 35977033 10.1111/birt.12674

[17] Jardim DMB, Modena CM (2018) Obstetric violence in the daily routine of care and its characteristics. Rev Lat Am Enfermagem 26:1–12. 10.1590/1518-8345.2450.3069 10.1590/1518-8345.2450.3069 PMC6280177 30517571

[18] Aktaş S, Aydın R (2019) The analysis of negative birth experiences of mothers: a qualitative study. J Reprod Infant Psychol 37:176–192. 10.1080/02646838.2018.1540863 30375236 10.1080/02646838.2018.1540863

[19] Çalik KY, Karabulutlu Ö, Yavuz C (2018) First do no harm—interventions during labor and maternal satisfaction: a descriptive cross-sectional study. BMC Pregn Childbirth 18:1–10. 10.1186/s12884-018-2054-0 10.1186/s12884-018-2054-0 PMC6201531 30355293

[20] Matsuo K, Shiki Y, Yamasaki M, Shimoya K (2009) Use of uterine fundal pressure maneuver at vaginal delivery and risk of severe perineal laceration. Arch Gynecol Obstet 280:781–786. 10.1007/s00404-009-1015-2 19263062 10.1007/s00404-009-1015-2

[21] Pan H-S, Huang L-W, Hwang J-L, Lee CY, Tsai Y-L, Cheng W-C (2002) Uterine rupture in an unscarred uterus after application of fundal pressure. A case report. J Reprod Med 47:1044–1046 12516327

[22] Sartore A, de Seta F, Maso G, Ricci G, Alberico S, Borelli M, Guaschino S (2012) The effects of uterine fundal pressure (Kristeller maneuver) on pelvic floor function after vaginal delivery. Arch Gynecol Obstet 286:1135–1139. 10.1007/s00404-012-2444-x 22752555 10.1007/s00404-012-2444-x

[23] Mohamed HS, Elkheshen SA (2017) Effect of manual fundal pressure during the second stage of labor on maternal outcomes among parturient women. Am J Nurs Res 5:109–114. 10.12691/ajnr-5-4-2

[24] Shimada M, Suzuki S (2013) Clinical significance of deliveries with uterine fundal pressure maneuver at a single perinatal center in Japan. J Clincal Gynecol Obstet 2:10–14. 10.4021/jcgo87w

[25] Rodríguez-Almagro J, Hernández-Martínez A, Rodríguez-Almagro D, Quirós-García JM, Martínez-Galiano JM, Gómez-Salgado J (2019) Women’s perceptions of living a traumatic childbirth experience and factors related to a birth experience. Int J Environ Res Public Health. 10.3390/ijerph16091654 31085980 10.3390/ijerph16091654 PMC6539242

[26] Buhimschi CS, Buhimschi IA, Malinow AM, Kopelman JN, Weiner CP (2002) The effect of fundal pressure manoeuvre on intrauterine pressure in the second stage of labour. BJOG 109:520–526. 10.1111/j.1471-0528.2002.01399.x 12066941 10.1111/j.1471-0528.2002.01399.x

[27] Volkert A, Bach L, Hagenbeck C, Kössendrup J, Oberröhrmann C, Okumu M-R, Scholten N (2024) Obstetric interventions’ effects on the birthing experience. BMC Pregn Childbirth 24(1):1–8. 10.1186/s12884-024-06626-5 10.1186/s12884-024-06626-5 PMC11283698 39068395

[28] Kemper M (2014) Kristeller Handgriff: Aktueller Forschungsstand. Die Hebamme 27:228–231

[29] Peyman A, Shishegar F, Abbasi Z (2011) Uterine fundal pressure on the duration of the second stage of labor in Iran: a randomized controlled trial. J Basic Appl Sci Res 1:1930–1933

[30] Pinar S, Karaçam Z (2018) Applying fundal pressure in the second stage of labour and its impact on mother and infant health. Health Care Women Int 39:1–32. 10.1080/07399332.2017.1376332 28956720 10.1080/07399332.2017.1376332

[31] Kanninen T, Bellussi F, Berghella V (2022) Fundal pressure to shorten the second stage of labor: systematic review and meta-analysis. Eur J Obstet Gynecol Reprod Biol 275:70–83. 10.1016/j.ejogrb.2022.06.010 35753230 10.1016/j.ejogrb.2022.06.010

[32] Okafor UB, Cheryl VN, Singata-Madliki M (2018) Exploring women’s experience of uterine fundal pressure during second stage of labour at Midwives-led Obstetric Unit (MOU) in East London, South Africa. Afr J Phys Act Health Sci 24:72–92

[33] Tong A, Sainsbury P, Craig J (2007) Consolidated criteria for reporting qualitative research (COREQ): a 32-item checklist for interviews and focus groups. Int J Qual Health Care 19:349–357. 10.1093/intqhc/mzm042 17872937 10.1093/intqhc/mzm042

[34] Okumu M-R, Bach L, Haid-Schmallenberg L, Karbach U, McKee L, Stevens NR et al (2024) Women’s experience of obstetric violence: a grounded theory study of perception formation during childbirth. Authorea Preprints. 10.22541/au.172114952.29794585/v1

[35] Corbin J, Strauss A (2015) Basics of qualitative research: techniques and procedures for developing grounded theory, 4th edn. SAGE Publications Inc., Thousand OAks

[36] Guest G, Namey E, Chen M (2020) A simple method to assess and report thematic saturation in qualitative research. PLoS ONE 15(5):1–17. 10.1371/journal.pone.0232076 10.1371/journal.pone.0232076 PMC7200005 32369511

[37] Themelis C, Sime J-A, Thornberg R (2022) Informed grounded theory: a symbiosis of philosophy, methodology, and art. Scand J Educ Res 67:1086–1099. 10.1080/00313831.2022.2115135

[38] Thornberg R (2012) Informed grounded theory. Scand J Educ Res 56:243–259. 10.1080/00313831.2011.581686

[39] Murphy DJ, Strachan BK, Bahl R (2020) Assisted vaginal birth: green-top guideline no. 26. BJOG 127:e70–e112. 10.1111/1471-0528.16092 32346983 10.1111/1471-0528.16092

[40] Häggsgård C, Nilsson C, Teleman P, Rubertsson C, Edqvist M (2022) Women’s experiences of the second stage of labour. Women Birth 35:e464–e470. 10.1016/j.wombi.2021.11.005 34872874 10.1016/j.wombi.2021.11.005

[41] Raoust GM, Bergström J, Bolin M, Hansson SR (2022) Decision-making during obstetric emergencies: a narrative approach. PLoS ONE 17(1):1–21. 10.1371/journal.pone.0260277 10.1371/journal.pone.0260277 PMC8791468 35081113

[42] Mirzania M, Shakibazadeh E, Bohren MA, Hantoushzadeh S, Babaey F, Khajavi A, Foroushani AR (2023) Mistreatment of women during childbirth and its influencing factors in public maternity hospitals in Tehran, Iran: a multi-stakeholder qualitative study. Reprod Health 20:1–14. 10.1186/s12978-023-01620-0 10.1186/s12978-023-01620-0 PMC10207711 37226263

[43] Baranowska B, Pawlicka P, Kiersnowska I, Misztal A, Kajdy A, Sys D, Doroszewska A (2021) Woman’s needs and satisfaction regarding the communication with doctors and midwives during labour. Deliv Early Postpartum Healthcare (Basel) 9(4):1–10. 10.3390/healthcare9040382 10.3390/healthcare9040382 PMC8066117 33915688

[44] Brandão T, Cañadas S, Galvis A, de Los Ríos MM, Meijer M, Falcon K (2018) Childbirth experiences related to obstetric violence in public health units in Quito, Ecuador. Int J Gynaecol Obstet 143:84–88. 10.1002/ijgo.12625 30025157 10.1002/ijgo.12625

[45] Simkin P (2017) Should ACOG support childbirth education as another means to improve obstetric outcomes? Response to ACOG committee opinion # 687: approaches to limit intervention during labor and birth. Birth 44:293–297. 10.1111/birt.12306 28971514 10.1111/birt.12306

[46] O’Brien D, Butler MM, Casey M (2017) A participatory action research study exploring women’s understandings of the concept of informed choice during pregnancy and childbirth in Ireland. Midwifery 46:1–7. 10.1016/j.midw.2017.01.002 28092814 10.1016/j.midw.2017.01.002

[47] Bringedal H, Aune I (2019) Able to choose? Women’s thoughts and experiences regarding informed choices during birth. Midwifery 77:123–129. 10.1016/j.midw.2019.07.007 31323487 10.1016/j.midw.2019.07.007

