# Supplementary material for: Making sense of fundal pressure: A qualitative study on women’s experiences of a non-evidence-based yet commonly practiced intervention
Source: Arch Gynecol Obstet. 2025 Jul 24;312(4):1277–86. doi: 10.1007/s00404-025-08130-3 (PMC12414011; doi:10.1007/s00404-025-08130-3)
Supplement: Supplementary file 1 — Supplementary file2 (PDF 482 KB) [file 404_2025_8130_MOESM1_ESM.pdf]

Summary of interview guides[1]

1. Interview guide – Experience of obstetric violence

Narrative prompt:
I now ask you to think about the birth of your child/children and tell me about the violent aspects.

- Follow-up questions referred to:
  - What exactly was perceived as violent
  - Relationship with care givers, communication and decision-making
  - What should have been done differently
  - Perceived autonomy and control

Narrative prompt:
Now that we talked about your birth experience, I ask you to tell me about your thoughts and feelings during pregnancy when anticipating the birth of your child.

- Follow-up questions referred to:
  - Wishes and hopes
  - Expectations
  - Fulfillment of expectations

Closing part:
  - Reflections directed to the care givers
  - Additions

2. Interview guide – Experience of fundal pressure

Narrative prompt:
I now ask you to think about the birth of your child/children and tell me about the part of the birth in which the fundal pressure was applied.

- Follow-up questions referred to:
  - Mode of applying fundal pressure
  - Emotional and physical experience during the intervention
  - What exactly was perceived as helpful/pleasant or violent/negative (depending on the narration)
  - Relationship with care givers: Communication and decision-making
  - In case of negative experience: What should have been done differently
  - Perceived autonomy and control during the intervention
  - Relationship with care givers: Emotions

Narrative prompt:
Now that we talked about your fundal pressure experience, I ask you to tell me how you experienced the other stages of labour in the delivery room.

---

[1] The original interview guides are available here: Okumu M-R, Bach L, Haid-Schmallenberg L, Karbach U, McKee L, Stevens NR, et al. Women's Experience of Obstetric Violence: A Grounded Theory Study of Perception Formation During Childbirth [Preprint] 2024. doi:10.22541/au.172114952.29794585/v1

- Follow-up questions referred to:
    - Other aspects of the birth experience that were perceived as violent, bad, helpful, positive etc. (depending on the narration)
    - Feeling before the application of fundal pressure
    - Relationship with care givers
    - Perceived autonomy, self-efficacy and control

Narrative prompt:
Now that we talked about your birth experience, I ask you to tell me about your thoughts and feelings during pregnancy when anticipating the birth of your child.

- Follow-up questions referred to:
    - Wishes and hopes
    - Expectations
    - Fulfillment of expectations
